# Establishment and Benefits of a Normal Pressure Hydrocephalus Support Group on Patient Education and Experience

**DOI:** 10.7759/cureus.5007

**Published:** 2019-06-26

**Authors:** Peter Tran, Christopher Q Nguyen, Melissa Huang, Judy Pham, Catthi Ly, Ishan Shah, Ronald Sahyouni, Cassie Poole, Kieu Tran, Jefferson W Chen

**Affiliations:** 1 Neurosurgery, University of California, Irvine Medical Center, Orange, USA; 2 Neurosurgery, University of California, Irvine School of Medicine, Orange, USA

**Keywords:** normal pressure hydrocephalus (nph), support group, likert-type, interactive learning, ibooks

## Abstract

Introduction: Normal pressure hydrocephalus (NPH) is a debilitating, neurological condition that can lead to mental deterioration. With the diagnosis and treatment of NPH constantly evolving and its symptoms worsening with age, education for patients and their families is crucial. In this study, we aim to explore the potential educational benefits of a physician-led NPH support group.

Methods: Between December 2015 and November 2018, six semiannual NPH support group meetings were held for patients and their families. Attendees, ages 20-90, completed a Likert scale-based survey designed to assess the support group’s educational benefits using the following primary outcome variables: (1) subjective knowledge, (2) perceived utility/efficacy, and (3) patient satisfaction.

Results: Our survey data suggests that patients and their family members agree on the efficacy of the support group in learning about NPH. They felt that the support group served its purpose and improved their comfort/knowledge regarding NPH. There was consensus about sustaining and maintaining the support group for the future. Of 65 survey responses, the composite average score of questions pertaining to subjective knowledge, perceived utility/efficacy, and patient satisfaction was 4.5 out of 5.0.

Conclusion: We demonstrated that support groups are effective in educating the adult NPH population and their family/friends about NPH onset and treatment. Enhanced educational awareness for patients and families can help patients cope with their neurological condition and improve patient adherence to follow-up and physician recommendations.

## Introduction

Normal pressure hydrocephalus (NPH) is a neurological condition characterized by the classic triad of gait disturbance, urinary incontinence, and memory dysfunction, as well as ventriculomegaly which can lead to mental deterioration if left untreated [1-2]. In the United States, the prevalence of NPH is estimated to be about 700,000 [3]. As patients age, the incidence becomes higher [4-6]. The diagnosis and treatment of NPH is rapidly undergoing continuous evolution [7-9]. This leads to confusion among patients and their families about prospective treatments and prognosis after the initial diagnosis. Consequently, education for patients and their families is important. Previous studies have demonstrated that patients' education is crucial in augmenting their compliance to physician recommendations [10-12]. This is particularly pertinent within the NPH cohort, in which some patients are treated with conservative management while others undergo surgery with the placement of a shunt.

Medical support groups are collaborative meetings among patients diagnosed with a common condition to educate and guide them through their experience [11]. Past literature has shown that support groups improve attendees’ overall knowledge of their condition as well as increase their adherence to medical treatment plans which may lead to improved health [11]. Additionally, patients reported improved decision making and lower levels of depression and anxiety [13-14]. For example, Leavitt et al. found that their brain tumor support group was beneficial in providing a therapeutic forum and emotional support for patients and their family members [15].

There are several organizations in the United States directed at supporting patients with hydrocephalus such as the Hydrocephalus Association. However, there has been no published report of an NPH support group thus far. We conducted a support group for the last three years, at our medical center, for patients with NPH and their families to address patients’ need for education and support.

For this study, we investigated whether a semiannual support group for NPH patients and their families was beneficial as determined by the following primary outcomes: (1) subjective knowledge, (2) perceived utility/efficacy, and (3) patient satisfaction.

## Materials and methods

The semiannual NPH support group was conducted at a tertiary care academic medical center. Each meeting was hosted in English during a three-year time period. The first meeting was held in November 2015 and the last was held in December 2018, with plans to continue semiannually. Support group meetings were organized by a volunteer staff that consisted of physicians, physical and occupational therapists, physician assistants, medical students, undergraduates, and other interested community members.

**Figure 1:**
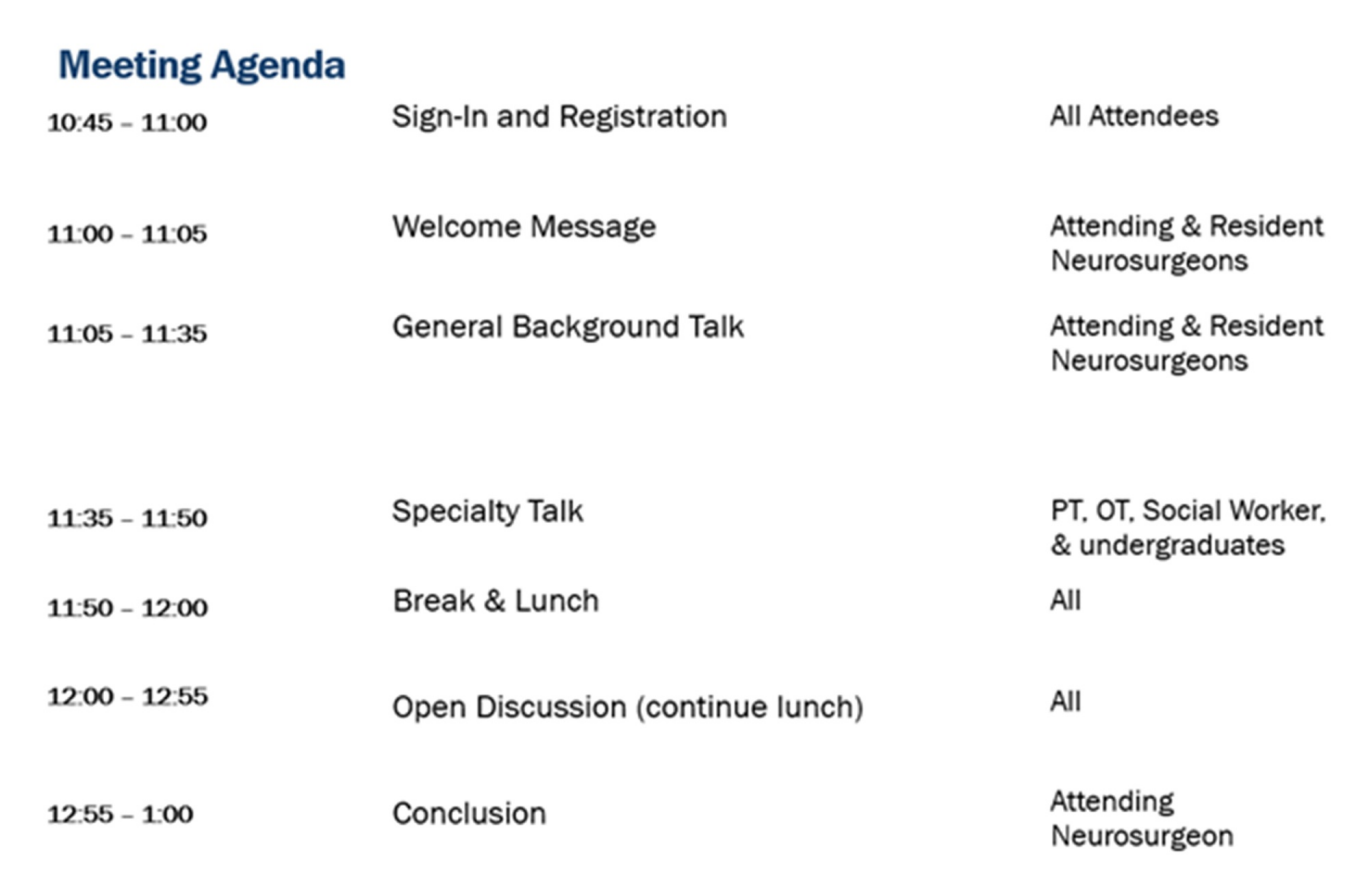
General outline of each meeting PT: patients; OT: occupational therapists.

Each meeting featured brief physician-led presentations about NPH, followed by lunch, and then a round-table discussion. Figure 1 provides a general outline of each meeting. Therapists and social workers were invited to give patients and their families their perspectives about NPH. In addition, patients and their families were also introduced to the interactive NPH iBooks which were preloaded onto an Apple® iPad (Apple Inc., Cupertino, California). Attendees were taught how to access the iBooks for free through the online Apple® Book Store [16].

Participants included NPH patients and their family members. Patients included those who were recently diagnosed with NPH and those who already had surgical intervention and wanted to share their perspectives with others. Patients were informed of the support group during clinic visits and via word-of-mouth.

**Figure 2:**
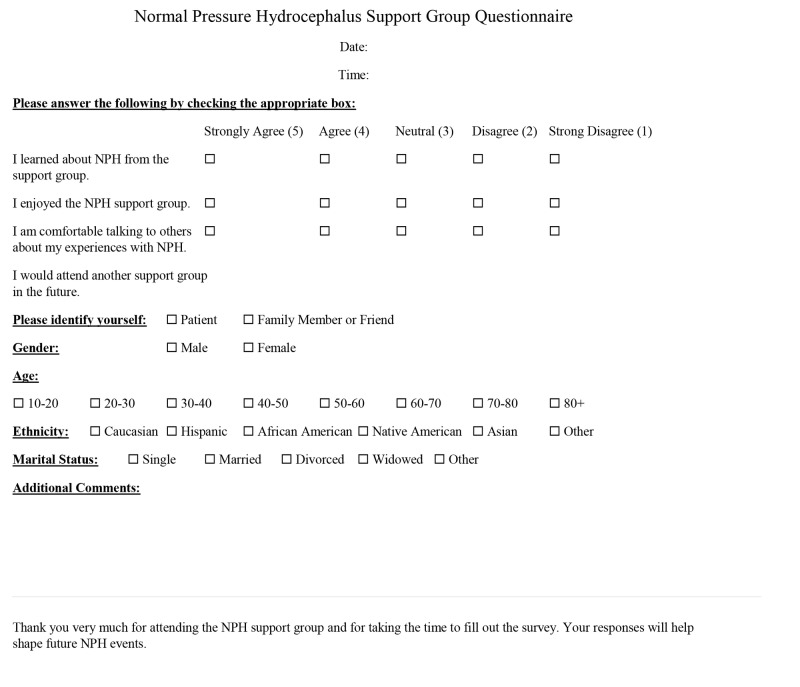
Survey taken by each participant NPH: normal pressure hydrocephalus.

At the end of each support group, attendees were asked to complete a Likert scale-based post-survey to assess the efficacy of the support group (Figure 2). The survey responses used a 5-point Likert scale to gauge the participants’ agreement with each statement: strongly agree (5), agree (4), neutral (3), disagree (2), and strongly disagree (1) [17]. The average survey score of all 4 questions (n = 65) was calculated for each subgroup. An unpaired two-tailed t-test was used to analyze significance (p < 0.05) between patients and family/friends.

## Results

Sixty-five attendees responded to the post-event survey. Of these, 35 were patients and 30 were family members or friends. The gender, age, ethnicity, and marital status of the participants are summarized in Table 1. The male to female attendee ratio is 1.09:1. The average age of the attendees, calculated based on reported age bracket, is 66 years old with an age range of 20-90 years of age. The average age of patients was 70 years old while the average age of family/friends was 61 years old. The majority of the participants (79%) are either married or had a long-term partner, whereas 21% are either single, divorced, or widowed. Additionally, 77.1% of participants are Caucasian, followed by 14.6%, 6.2%, and 2.1% for Asian, Hispanic, and mixed race, respectively.

**Table 1:** Demographics of participants *Discrepancies from total number of participants due to participants leaving these categories blank.

Total No. Participants (N = 65)		
Attendee	Patient (N = 35)	Friend/Family Member (N = 30)
Gender*	Male (N = 25)	Female (N = 23)
Marital Status	Married (N = 37)	
	Single (N = 4)	
	Divorced (N = 3)	
	Widowed (N = 3)	
	Long-term Partner (N = 1)	
	Not listed (N = 17)	
Race*	Caucasian (N = 35)	
	Asian (N =6)	
	Hispanic (N =3)	
	Mixed/Other (N = 1)	
Age Range	20-29 (N = 2)	
	30-39 (N = 2)	
	40-49 (N = 6)	
	50-59 (N = 7)	
	60-69 (N = 15)	
	70-79 (N = 23)	
	80+ (N = 9)	
	Not listed (N = 1)	

The first three support groups had a total of eight staff members (Table 2). Support groups 4 and 5 had a total of 12 staff members. Support group 6 had a total of 14 staff members.

**Table 2:** Number of staff members and volunteers per support group

Total No. Staff/Volunteers	
Support Group 1	8
Support Group 2	8
Support Group 3	8
Support Group 4	12
Support Group 5	12
Support Group 6	14

**Figure 3:**
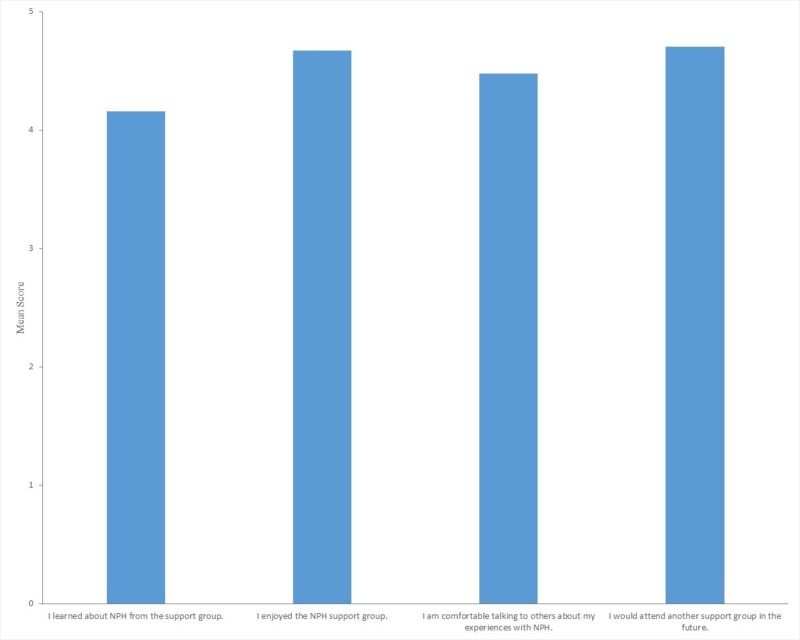
Average survey score of all attendees sorted by question

Figure 3 depicts the average scores for each statement. The first statement had an average response score of 4.14 ± 1.25, implying attendees felt that they learned about NPH from the support group. The second statement’s average response is 4.67 ± 0.76, indicating attendees enjoyed the support group. The third statement had an average of 4.48 ± 0.81, suggesting comfort in discussing their NPH experiences with others. The fourth statement had an average of 4.70 ± 0.66 suggesting that they would likely attend a future support group. Additionally, participants provided positive, constructive feedback via the additional comments section on the survey.

**Figure 4:**
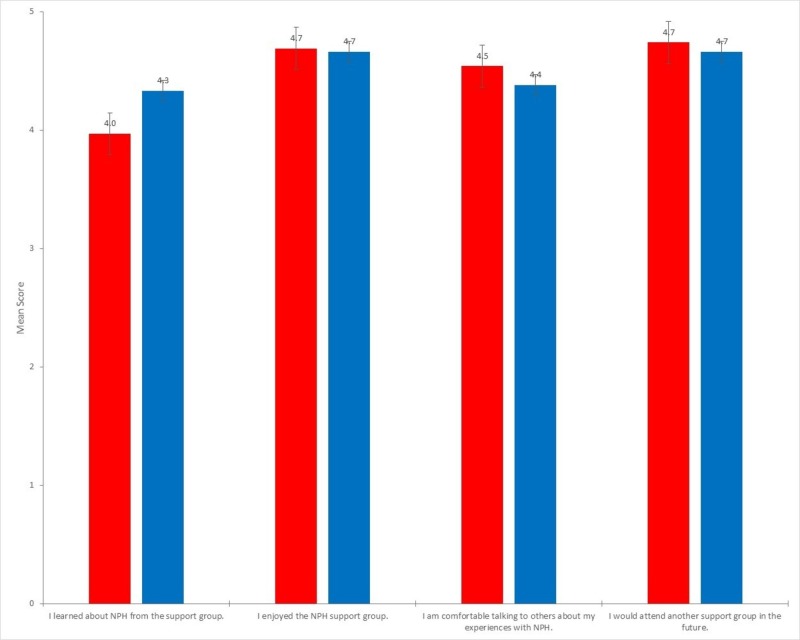
Average survey response score by question (patient vs. family) No statistical difference was found between patients and family/friends for each statement (p = 0.20, p = 0.87, p = 0.45, p = 0.60)

No significant difference was found when comparing the responses from patients and the responses from family members for statements 1 (p = 0.20), 2 (p = 0.87), 3 (p = 0.45), and 4 (p = 0.60), as shown in Figure 4.

## Discussion

For the three years in which we held the support group, the demographics remained constant. The majority of the NPH patients at our support groups identified as Caucasian. According to Bir et al., NPH is seen predominantly in Caucasian males in America [4]. However, other factors such as language barriers, referral patterns, and access to healthcare may have impeded the attendance of non-Caucasian NPH patients. 

This study demonstrated that the implementation of a semiannual support group enhanced patient and family understanding of NPH and was perceived efficacious as assessed by the subjective Likert scale post-surveys. Ultimately, the implementation of an NPH support group is meant to augment the management of NPH patients especially for those who undergo ventriculoperitoneal shunt placement. These patients require follow-up and education to help them recognize potential problems such as postoperative complications and symptomatic progressions. The round-table discussions were particularly helpful in allowing patients/families to share and compare their experiences. This allowed them to better appreciate the myriad of symptoms and responses to treatment that exist.

Overall, patients appeared to benefit from the NPH event and were interested in attending future support group meetings. As shown in Figure 3, survey responses indicated satisfaction with the event. Our data suggests that the physician-led, multi-modal support group was able to effectively educate patients and their families. The positive responses, especially about the interactive nature, from both patients and their families, demonstrate that the support group successfully addressed an audience with varying understandings of NPH that included NPH patients with potential cognitive limitations.

In addition, our support group differed from others in the literature because of the inclusion of the iBook. The iBook provides each NPH support group attendee with a more personalized experience and raises opportunities for thought-provoking questions. Past studies have demonstrated that these iBooks are effective in increasing patient understanding as well as providing comfort and assurance for those seeking surgical treatment [18-19]. Furthermore, they have been shown to be effective across multiple linguistic and cultural backgrounds and in geriatric patients, where technological usage could pose potential challenges that may interfere with the goals of the iBook [20-21]. The iBook also served as a resource for the patients, allowing the impact of the support group to last beyond the time allotted in the meetings.

Based on the comments and survey results, our NPH support group has evolved to cater to the needs and suggestions of attendees. Those who were likely to attend were patients who were contemplating treatment options and post-surgery patients who were strong advocates of shunt treatments.

We progressively incorporated additional methods of advertisement in each successive support group meeting. Earlier meetings were publicized by notifying patients during clinic visits. Flyers were circulated throughout the clinic to inform patients and family members of this opportunity. To increase patient turnout, those who expressed interest were reminded via email and phone call prior to each meeting.

In addition, the specialty talks have varied throughout different support groups to accommodate different guest speakers. Guest speakers have included physical therapists, occupational therapists, and social workers.

As our support group evolved, less time was dedicated for lectures and presentations and more time was dedicated for discussions since patients and their families enjoyed sharing and hearing personal experiences. This is especially helpful since patients who have undergone longer treatment and management of their NPH were able to advise and support newly diagnosed patients.

Although efforts were made to ensure the validity and accuracy of our results, this study has inherent limitations due to its study design. Because we utilized a Likert scale, there was no objective assessment of the patients’ and their families’ knowledge and understanding of NPH. Furthermore, with 65 responses from attendees across all six meetings, future studies will increase the sample size. Nonetheless, this study still demonstrates that a support group was effective in educating NPH patients and their families about the condition. However, an assessment of objective knowledge is still warranted.

## Conclusions

To our knowledge, this support group hosted at our medical center is one of the few active support groups specifically for NPH. The need for a support group for patients with NPH was brought about by the lack of public awareness about NPH. With the establishment of this semiannual support group, we have concluded that access to an NPH support group can be beneficial for patients and their family members. Our study shows that our support group was successful in improving patient education and comfort with NPH. Our NPH support group serves as a model that can be reproduced by others. The high satisfaction with the low-cost, interactive support group model, demonstrates its efficacy as a supplemental resource for patients.
